# Liver Oxidative Stress after Renal Ischemia-Reperfusion Injury is Leukocyte Dependent in Inbred Mice

**Published:** 2011

**Authors:** Hossein Khastar, Mehri Kadkhodaee, Hamid reza Sadeghipour, Behjat Seifi, Jamshid Hadjati, Atefeh Najafi, Manoocher Soleimani

**Affiliations:** 1 *School of Medicine, Shahroud University of Medical Sciences, Shahroud, Iran*; 2 *Department of Physiology, School of Medicine, Tehran University of Medical Sciences, Tehran, Iran*; 3 *Department of Immunology, School of Medicine, Tehran University of Medical Sciences, Tehran, Iran*; 4 *Department of Medicine, University of Cincinnati, MSB G259 OH, United States*

**Keywords:** Leukocyte, Liver, Oxidative stress, Renal ischemia

## Abstract

**Objective(s):**

There are some reports in recent years indicating that renal ischemia – reperfusion (IR) induces deleterious changes in remote organs such as liver. The aim of this study was to investigate whether leukocytes have a role on the induction of oxidative stress in liver after renal IR.

**Materials and Methods:**

Inbred mice in IR donor group were subjected to renal IR injury. In sham donor group the procedure was almost the same except that ischemia was not induced. Then, mice were anesthetized and blood was collected. Leukocytes were isolated from donor groups and were then transferred to intact recipient mice (from IR donor mice to IR recipient mice and from sham donor mice to sham recipient mice).

**Results:**

After 24 hr, hepatic superoxide dismutase (SOD) and catalase (CAT) activities decreased significantly in recipient mice that received leukocytes from IR donor mice in comparison to recipient mice received leukocytes from sham donor mice.

**Conclusion:**

These findings indicate that leukocytes are one of the mediators that induce hepatic oxidative stress after renal IR.

## Introduction

Acute kidney injury (AKI) is a common complication that occurs in some of hospitalized patients especially in intensive care units. Renal ischemia-reperfusion (IR) is one of the most important causative mechanisms of AKI (1, 2). Leukocytes infiltration and inflammatory responses are among the suggested causes of renal IR injury. Activation and migration of leukocytes into the kidney have been demonstrated in renal IR injury. These leukocytes produce reactive oxygen metabolite (ROM) (3-5). 

High mortality rate during AKI is largely due to remote organ dysfunction. Renal IR injury may also lead to the failure of other systems like lung, brain and liver (1, 2). Humoral or cellular factors are thought to be the causes of remote organ failure but their exact pathophysiological mechanisms are not completely understood (6, 7).

Previous studies have shown that AKI causes an increase in leukocyte infiltration in remote organs such as the liver. It has also been demonstrated that renal IR induces oxidative stress in liver causing its dysfunction. Renal IR caused an increase in hepatic tumor necrosis factor levels, myeloperoxidase activities and thiobarbituric acid reactive substance (TBARS) concentrations (4, 8). During renal IR, liver functional indices such as blood aspartate aminotransferase (AST) and alanine aminotransferase (ALT) were elevated and spermine - spermidine acetyl transferase, an enzyme up-regulated in early phases of hepatic injury, was increased (2). 

Superoxide dismutase (SOD) and catalase are the most important enzymatic antioxidant systems in the body. SOD, as the first and most important line of defense against reactive oxygen metabolites (ROM), transforms superoxide ion to H_2_O_2_ that is a less reactive molecule (9). CAT is a regulator of H_2_O_2_ metabolism and converts H_2_O_2_ into water. It is a tetrameric haemin-enzyme consisting of four identical tetrahedral arranged subunits (10). The aim of the present study was to investigate whether leukocytes have a role on the induction of oxidative stress in liver after renal IR. To do so, after induction of renal IR injury in donor mice, their leukocytes were transferred to intact recipient mice. Then the antioxidant status of liver in recipient mice was evaluated. 

## Materials and Methods

***Animal model of renal IR***

Male BALB/c mice (weight 25-35g) were randomly assigned to four groups (n= 9):

1- Donor mice that were undergone renal IR condition (IR donor),

2- Donor sham–operated control mice (Sham donor),

3- Recipient mice that received leukocytes from the first group (IR recipient),

4- Recipient mice that received leukocytes from the second group (Sham recipient). 

Mice in IR donor group were anesthetized with intraperitoneal pentobarbital sodium injection. Systolic blood pressure was measured by the tail-cuff method connected to a pneumatic transducer using a PowerLab/4sp data acquisition system (software Chart, version 5, AD Instruments, ). To perform ischemia, after midline incision, the renal pedicles were bluntly dissected and non-traumatic vascular clamps were applied. Ischemia was confirmed visually by renal blanching. Animals were received 60 min of ischemia followed by 3 hr of reperfusion. 

After reperfusion, clamps were removed gently and the kidneys were observed for a further 5 min to ensure reflow process. Then, 1 ml of sterile saline (37 °C) was injected intraperitoneally and the incision was closed in two layers with a 4-0 silk suture. The animals were then returned to their cages and allowed to recover. During the period of renal ischemia, the animals were covered with plastic wrap to prevent evaporation. In addition, animals were kept well hydrated with warm sterile saline and were maintained at a constant body temperature (~37 °C) on a heating pad. 

At the end of the reperfusion period, the animals were anesthetized. Blood samples were obtained from heart with heparinized syringes to avoid from clotting before WBC isolation. The abdomen was opened immediately and liver tissues were collected and were snap frozen. Tissues were maintained at -70 °C till enzyme evaluation. Care should be taken during all procedures including leukocyte isolation to be precise and fast.

Briefly, RBCs were removed from collected blood by lysis in NH_4_Cl. In this method NH_4_Cl lyses RBCs while WBCs were remained intact. The solution was centrifuged and supernatant that include WBCs was isolated. Isolated leukocytes were washed twice with phosphate-buffered saline (PBS), mixed with saline and counted using the trypan blue exclusion technique. Leukocytes (5×10^6^ cells) from donor groups were transferred to intact recipient groups of animals via the tail vein. After 24 hr, recipient mice were anesthetized and blood samples were obtained from heart with heparinized syringes and centrifuged at 4000 g for 10 min at 4^ ◦^C. Plasma samples were collected for biochemical analysis.

***Measurement of liver oxidative stress indices***

Hepatic SOD activity was determined by the method of Paoletti and Mocali (11). In this method superoxide anions are generated from oxygen molecules in the presence of EDTA-MnCl_2_ and mercapto-ethanol. NAD (P)H oxidation is linked to the availability of superoxide anions in the medium. As soon as SOD is added to the assay mixture, it inhibits nucleotide oxidation. Therefore, at high concentration of the enzyme the absorbance at 340 nm remains unchanged. 

For this purpose, 50 mg liver tissues were hemogenized in 500 µl phosphate buffer. After 20 minutes the mixture was centrifuged in 4200 RPM, 400 µl supernatant was added to 1 ml phosphate buffer, and then was inserted in dialysis tubes inside the phosphate buffer for 15-18 hours (4 ^◦^C). The following solutions were subsequently added into the cuvette: 0.8 ml triethanolamine-diethanolamine-HCl buffer, 40 µl NADPH solutions, 25 µl EDTA-MnCl_2_ and 100 µl from different samples. In the 5th minute mercaptoethanol was also added. Absorbance changes were detected at 340 nm. For the calculation, the following equation was used:

Sample rate/control rate×100 = % inhibition

Then the concentration of enzyme was obtained in Paoletti’s standard table. 

CAT activity was determined by Aebi’s method (10). According to this method, activity of CAT can be measured by decomposition of H_2_O_2_. The remaining substrate concentration at a given moment of the reaction can be determined by UV spectrophotometry at 240 nm.

For this purpose, 50 mg liver tissues were homogenized in 450 µl isotonic buffer and 50 µl triton (%1). After 15 minutes the mixture was centrifuged in 4200 RPM and then the supernatant was diluted 500 times with phosphate buffer. Then 2 ml of this solution was added to 1 ml of hydrogen peroxide. This was monitored for 30 seconds at 240 nanometer by spectrophotometery. Enzyme activity was calculated using the following equation:

[K= (2.3/30) × (logA1/A2) a]

in which A1 is absorbance at time zero and A2 is absorbance at second 30 (a is dilution factor).

***Statistical analysis***

Means and standard deviations were calculated. Unpaired student t-test was used for comparison, and statistical significance was determined as *P*< 0.05.

## Results

***Effect of renal IR injury on liver oxidative stress indices***

Renal IR injury led to decreases in liver SOD and CAT activities (*P*= 0.016 and *P*= 0.007, respectively) in IR donor mice compared to sham donor. IR recipient mice also showed reductions in liver SOD and CAT activities (*P*= 0.020 and *P*= 0.025, respectively) compared to sham recipient.

**Figure 1.**
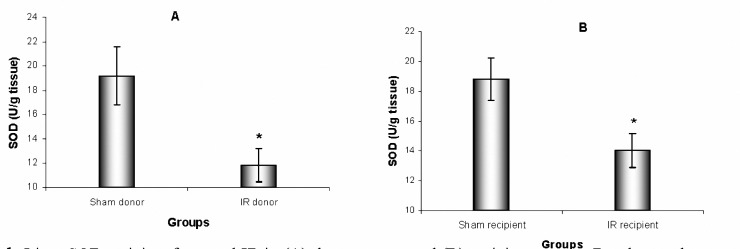
Liver SOD activity after renal IR in (A) donor groups and (B) recipient groups. Results are the mean±SEM (* *P*< 0.05 by *t*-test compared with sham, n= 9)

**Figure 2.**
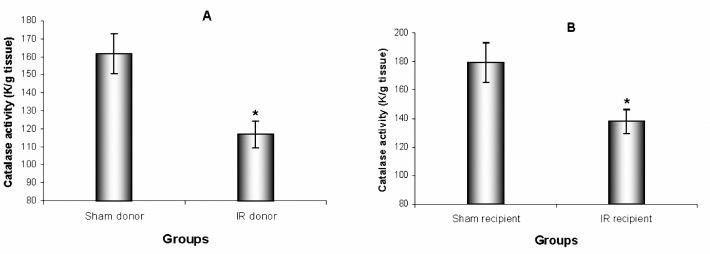
Liver CAT activity after renal IR in (A) donor groups and (B) recipient groups. Results are the mean±SEM (* *P*< 0.05 by t-test compared with sham, n= 9)

## Discussion

Renal IR damage is one of the most common causes of AKI. Ischemia-reperfusion is a serious problem that affects the outcome of various surgical operations such as organ transplantation and surgical revascularization. When AKI is associated with multiple organ failure causes a high mortality rate (1,6). 

This study represents a renal IR model which induces liver oxidative stress. We evaluated hepatic SOD and CAT activities as indices of oxidant – antioxidant status of liver tissues. After 60 min of ischemia followed by 3 hr of reperfusion, significant decreases in liver SOD and CAT activities were seen. These finding confirmed that renal IR ischemia induces induction of liver oxidative stress. In IR recipient group that did not subjected to any ischemia and reperfusion, significant decreases in liver SOD and CAT activities were also seen. This suggests that leukocytes may have a role by themselves in the induction of hepatic oxidative stress. 

Superoxide dismutase and catalase are the first line of tissue defense against oxidant substances that release from cells such as WBCs. Other studies have shown SOD and CAT levels were decreased in conditions such as oxidative stress (12). It has been demonstrated that leukocytes have a role in the local organ IR injury after induction of reperfusion injury (5). Activated neutrophils adhere to vascular endothelial cells in the vasa recta of the outer medulla of the kidneys. In addition, activated neutrophils infiltrate into the tissues and release a variety of inflammatory cytokines and enzymes, leading to tissue injury (12).

It has been demonstrated by Rabb *et al* in 2000 that mice deficient in CD4^+ ^and CD8^+^ T cells had less renal IR injury. Serum creatinine was reduced at 24 hr after renal IR in CD4^+ ^T cells deficient mice compared to control mice with renal IR. They concluded that T cells are important mediators of ischemic AKI. CD4/CD8 deficient mice had a markedly decreased neutrophil infiltration into renal tissue after IR compared to wild type control mice. This observation suggests that T cells have a role in organ IR injury (3). In other studies, it was shown that B cell-deficient mice had a reduced renal IR injury (13).

Renal IR induces remote organ injury in addition to kidney injury. Kelly (2003) showed that renal IR injury elevates leukocyte infiltration in heart, liver and lung. In that study, increased IL-1 and intercellular adhesion molecule-1 mRNA and elevated immunoreactive TNF-alpha levels were observed in the heart after renal ischemia in the rat (6).

Serteser *et al*. showed that renal IR injury induces liver oxidative stress. They evaluated catalase and superoxide dismutase activities, total glutathione concentration, TBARS and protein carbonyl levels (4). In addition, their study as well as Golab *et al* study, showed an increase in hepatic TNF-α levels (2,4). 

In 2006, Emre *et al* showed that renal IR injury led to an increase in liver SOD and CAT activity but GPx and TBARS did not show significant changes in the same rat. They concluded that 30 min ischemia followed by 2 hr reperfusion may not be enough to change GPx and TBARS (14).

In the present study, transfer of activated leukocytes caused a significant level of oxidative stress, namely a decrease in superoxide dismutase and catalase activities, in the liver of recipient mice that received leukocytes from renal IR mice in comparison to recipient mice received leukocytes from sham-operated control mice. 

## Conclusion

These findings indicate that leukocytes are one of the mediators that induce hepatic oxidative stress after renal IR.
